# Design and Experimental Verification of a Gibbon-Inspired Tree-Climbing Robot for Forestry Environments

**DOI:** 10.3390/biomimetics11050332

**Published:** 2026-05-09

**Authors:** Xinzhe Lu, Jianshuo An, Latai Ga, Xiaopeng Bai, Daochun Xu, Wenbin Li

**Affiliations:** 1 School of Technology, Beijing Forestry University, Beijing 100083, China; luxinzhe22@bjfu.edu.cn (X.L.); anjianshuo@bjfu.edu.cn (J.A.); xiaopengbai@bjfu.edu.cn (X.B.); xudaochun@bjfu.edu.cn (D.X.); 2 Key Laboratory of National Forestry and Grassland Administration on Forestry Equipment and Automation, Beijing 100083, China; 3 School of Renewable Energy, Inner Mongolia University of Technology, Ordos 017010, China; galtai@imut.edu.cn; 4 Inner Mongolia Academician Workstation for New Energy Intelligent Equipment and Operation Maintenance, Ordos 017010, China

**Keywords:** tree-climbing robot, bionic design, series–parallel structure, kinematic analysis, simulation verification, control system, field experiments

## Abstract

Tree-climbing robots are primarily utilized for pruning and harvesting in tall trees; however, limited structural degrees of freedom (DoFs) reduce their flexibility in complex environments. To improve the flexibility and environmental adaptability of the robots, this study proposes a novel three-armed claw-type tree-climbing robot inspired by gibbons. A 14 DoFs prototype with a total mass of approximately 2.52 kg was developed, comprising three manipulator arms and independently actuated claws. Kinematic models were separately established for the series-connected arms and the parallel-connected moving platform, with accuracy verified through numerical simulations. Based on these models, a control system was implemented, and a physical prototype was tested in field climbing experiments. Grasping tests on surfaces of varying roughness, including moist tree trunks, artificial wood, and smooth steel plates, demonstrated the adaptability of the claw to diverse materials. The robot successfully climbed trunks inclined at 52–90°, supporting a maximum payload of 1.81 kg; each full gait cycle averaged approximately 4 min. These results indicate that the robot can successfully imitate the movements of gibbons during climbing, thereby verifying the feasibility and practical application value of this bionic design in real-world forestry environments.

## 1. Introduction

In recent years, advances in fields such as robotics and computer science have led to the development of climbing robots capable of moving on vertical and irregular surfaces to perform specialized tasks [1,2]. Concurrently, the exceptional characteristics exhibited by natural organisms, including strong adaptability, robust locomotion capabilities, and multifunctionality [3], are driving biomimetic design as a key approach to enhancing the adaptability of climbing robots in complex environments [4]. These robots have found widespread application in scenarios such as building maintenance [5,6], rescue at high altitudes [7], and the exploration of natural resources [8].

Compared to climbing robots designed for man-made structures such as walls and poles, tree-climbing robots are faced with more complex environments because trees are characterized by variable morphology, uneven branch diameter, and the presence of obstacles such as branches and galls [9]. Researchers have developed a variety of tree-climbing robots to meet the requirements of operating in complex tree environments [10]. According to the different types of contact between the robots and the tree trunk, the existing tree-climbing robots can be divided into three categories: wheeled, encircling, and clawed. Each type has specific application scenarios.

Wheeled tree-climbing robots use drive wheels as a propulsion mechanism to enable rapid movement along tree trunks. For example, Sebastian et al. designed a four-wheeled climbing robot that can traverse tree trunks with a slight inclination [11]. Ban et al. improved the reliability of the wheel–trunk contact by incorporating a crawler belt in their “Monkeybot”, which can quickly climb trees and trim branches [12]. To address the challenges of manual coconut harvesting, various wheeled harvesting robots have also been developed, such as an autonomous harvesting robot using rubber rollers [13] and a coconut harvester based on Mecanum wheels [14]. Overall, wheeled robots demonstrate high climbing efficiency on smooth and less branched trunks but are poor at avoiding obstacles such as branches. This makes them more suitable for pruning and harvesting tasks on tree species with uniform diameters and smooth surfaces.

Encircling climbing robots achieve clamping functionality through components that conform closely to the trunk, utilizing appropriate mechanisms to alternate between ascending and descending motions. For example, Ma et al. proposed a piezoelectric pole-climbing robot, the “Crabbot” [15], which consists of two clamping units and an extension unit, and the stepping displacements are amplified by the designed flextensional mechanism. Wang et al. designed a variable-cell robot that clamps the trunk using linkages in its feet and accomplishes movement through alternating legs [16]. After developing a flexible manipulator [17], Ito et al. built a bionic octopus-like multi-legged robot and performed climbing experiments in different scenarios [18]. Liao et al. proposed a rope-winding climbing robot [19]. It features two winding actuators as the head and tail and a telescopic actuator as the body, enabling rapid linear movement on tree trunks. Takemori et al. developed a flexible serpentine robot that autonomously adapts to the trunk diameter through spiral rolling motions [20], facilitating movement on tree surfaces. In general, encircling tree-climbing robots maintain close contact with the trunk, providing stability and safety during operation. However, their flexibility is reduced due to the obstruction of branches during the grasping process.

Clawed tree-climbing robots achieve grasping by hooking their claws into the bark or holding onto the trunk. These robots are often inspired by the structural features or climbing techniques of animals. Currently, claw-type tree-climbing robots can be classified according to their DoFs, with significant differences in efficiency, payload and adaptability. Claw-type robots with low DoFs (1–3 DoFs) typically rely on passive grasping mechanisms that hook onto the bark for lightweight design and environmental adaptability. For example, the “LORIS” robot has a novel micro-spiked claw that can latch onto bark protrusions, with a total weight of 3.2 kg [21]. Lam & Xu designed the “Treebot”, which uses two claws to hook onto the bark and incorporates three actuators as the main body structure to mimic the climbing posture of an inchworm [22,23], with a total weight of just 600 g. However, these robots are constrained by their gripping force and terrain dependency, making it difficult for the body to detach from the trunk and actively adjust its posture. They demonstrate insufficient adaptability when confronted with complex environments such as variations in trunk diameter, tree knots, or galls. Mid-DoF (4–6 DoFs) claw-type robots typically use a single manipulator arm for climbing, with claws at both ends for grasping. For example, Du et al. designed a 4 DoFs robotic arm that uses large claws to grip the tree trunk and developed a corresponding obstacle-crossing gait [24]. Guan et al. has developed a modular dual claw robot called “Climbot” [25,26], which can climb poles and trusses and also act as a manipulator arm for specific tasks. These designs extend the workspace through serial joints, but during motion, typically only one mechanical claw grips the trunk. This arrangement renders the robot prone to instability when its center of gravity shifts. High-DoF (greater than 6 DoFs) claw-type robots generally adopt a multi-legged parallel architecture. For example, the six-legged bionic robot “Rise”, developed by Spenko et al., can climb not only tree trunks but also rough brick and gravel walls due to its special foot structure [27]. Inspired by the longicorn and gecko, Bian et al. developed a four-legged climbing robot with small claws that can climb various complex surfaces such as smooth pipes and tree bark [28]. These robotic platforms provide high stiffness support through multiple serial mechanical legs, thereby avoiding the center-of-gravity instability inherent in single serial structures and exhibiting considerable load-bearing capacity. However, the distinctive leg structure of existing, high-DoF claw-type robots means their bodies require constant contact with the tree trunk, resulting in size constraints that limit their operational capabilities. Furthermore, structural redundancy complicates multi-legged control algorithms, increasing risks in practical applications.

In summary, wheeled and encircling tree-climbing robots are constrained by their propulsion mechanisms, rendering them more suitable for tall trees with smooth bark. In contrast, nature-inspired stepper-type clawed robots demonstrate stronger adaptability and obstacle avoidance capabilities [29]. Based on this, the core research problem proposed in this study is how to design a tree-climbing robot based on bionic principles with a simplified structure, efficient control, and environmental adaptability to solve the problems of insufficient climbing stability and limited obstacle avoidance capabilities of existing equipment in complex forestry scenarios.

Therefore, the research objective of this paper is to develop a novel three-armed claw-type tree-climbing robotic system based on bionic principles, thus achieving stable and efficient climbing in complex tree environments. The main contributions and innovations of this study are mainly reflected in three aspects: first, conducting biomimetic analysis and design based on the climbing characteristics of gibbons; second, ensuring stable gripping through at least two claws at all times while adopting a hybrid series–parallel structure for the robot, which reduces the complexity and energy consumption of the control system by minimizing redundant limbs and driving joints; third, enabling the robot body to actively detach from the tree trunk and adjust its posture through the coordinated movement of the manipulator arms, thereby significantly enhancing its obstacle avoidance capabilities in complex environments and solving the key issue of insufficient obstacle avoidance flexibility in existing robots. This study provides a novel technical solution for efficient and stable tree climbing and autonomous operation in forest environments, enriching the design ideas and technical paths of inspired tree-climbing robots.

The rest of the article is organized as follows: Section 2 introduces the biomimetic design approach and the overall structural modeling of the robot. Section 3 verifies the structural performance of the robot through numerical simulations, describes the fabrication of the robot prototype and the development of the control system, and presents the results of field experiments. Section 4 provides a discussion on the stability and motion performance of the prototype. Section 5 summarizes the research work and points out the potential improvement directions for the future.

## 2. Materials and Methods

### 2.1. Biomimetic Design of Tree-Climbing Robot

In the natural world, gibbons living in the tropical rainforests of eastern and south-eastern Asia exhibit exceptional arboreal abilities. They are adept at foraging and evading predators in complex forest environments, using a variety of supports for rapid climbing [30]. Studies have shown that gibbons have evolved specific traits that make them good climbers: elongated forelimbs, highly flexible joints, hook-like hands, and a weight bias towards the hindlimbs [31,32]. Qualitative observations reveal that gibbons can use their forelimbs to grip the trunk from the side or behind, while their robust hindlimbs are curled up to bear compressive loads and provide stable support [33]. During climbing, the torso of the gibbon stays almost out of contact with the trunk. The forelimbs search for gripping points while the hindlimbs generate upward thrust, enabling the gibbon to navigate nimbly and steadily over obstacles such as branches and galls [34].

**Figure 1:**
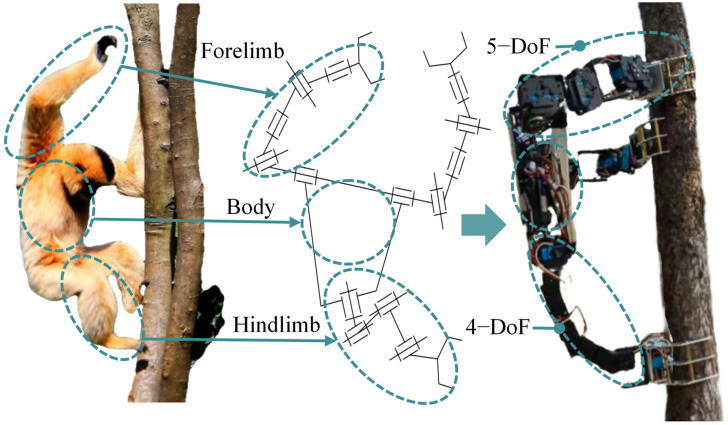
Design concept for the gibbon-inspired tree-climbing robot.

By analogy with gibbons, a series–parallel hybrid structure consisting of multiple tandem chains can perform a similar function. In a serial chain, components are arranged in a sequence to form a continuous link, allowing the end effector to move over a larger range, similar to the forelimb of the gibbon. When mechanical claws are used as the end effector, it enables the gripping action on the tree trunk. Parallel mechanisms consist of multiple serial chains connected in parallel. They offer advantages such as low inertia, high stiffness, and a large effective payload, making them suitable for bodies with moderate DoFs and high load-bearing requirements [35], similar to the gibbon torso. Therefore, a series–parallel hybrid mechanism is employed in the design to achieve stability and flexibility of the robot during tree climbing. For tree-climbing robots, since movement on flat ground is unnecessary, fully replicating the structure of the gibbon limb would result in higher manufacturing costs and mechanical redundancy. In order to reduce the complexity of the robot, a simplified design was adopted, with the design concept illustrated in Figure 1.

The forelimbs of gibbon have seven anatomical DoFs, and their behavior of grasping the trunk from the side or rear is irreplaceable; during climbing, the hindlimbs of gibbons, through coordinated movements of the hip and ankle joints, bear the dual functions of supporting and propelling the trunk [36]. This suggests that the hindlimbs are functionally replaceable in terms of mechanics. Moreover, the direction of force exerted by the hindlimb of primates on the tree trunk is generally consistent [37]; the hindlimbs both push against the trunk from the front to maintain overall body stability. This coordination allows the bionic design to simplify the hindlimb into a single functional unit, reduced to a mechanical arm placed along the central axis of the robot.

**Figure 2:**
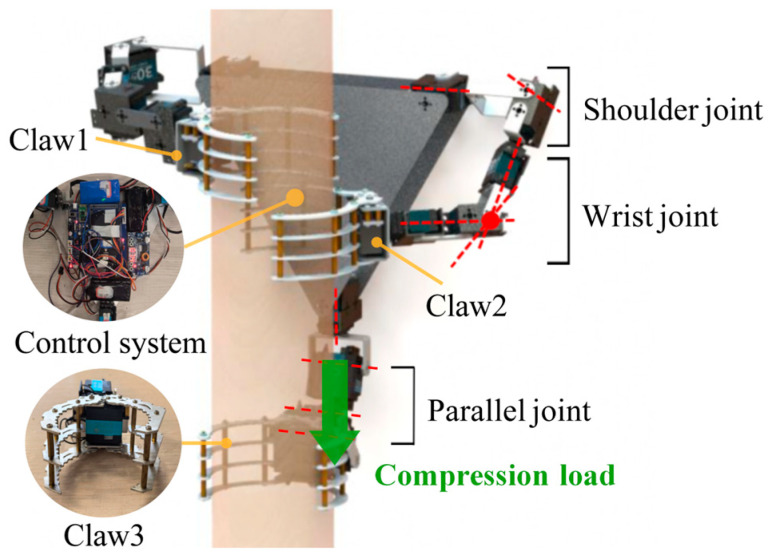
3D prototype modelling.

The overall structure of the designed gibbon-inspired tree-climbing robot is illustrated in Figure 2 as a triangular rigid platform equipped with three manipulator arms, with a total of 14 DoFs, with a mechanical claw at the end of each arm used to grasp the tree trunk. The two front manipulator arms are symmetrically distributed around the central axis of the body, and each has five degrees of rotational freedom, mimicking the elongated and flexible forelimbs of the gibbon. According to Pieper’s criterion to ensure that the manipulator arm is a closed-form solution [38], the three rotational axes near the end effector are intersected at the wrist point. Therefore, the single front manipulator arm has two DoFs at the shoulder joint and three collinear rotational axes at the wrist joint. This configuration enables the robot to adapt to tree trunks of varying shapes and inclination angles. The rear manipulator arm is equipped with a rotary joint aligned with the central axis of the body, which facilitates convenient adjustment of the overall body posture. Drawing on the force contraction and active extension mechanism of gibbons’ hindlimbs for power generation, the rear manipulator arm was simplified to four rotational DoFs. The three rotational axes near the claw are arranged parallel to one another so as to withstand compressive loads and deliver upward thrust, thereby simulating the function of the hindlimb of a gibbon [39]. The robotic body consists of two triangular aluminum alloy plates that secure the three manipulator arms and provide a mounting space for components such as the control boards and batteries.

**Figure 3:**
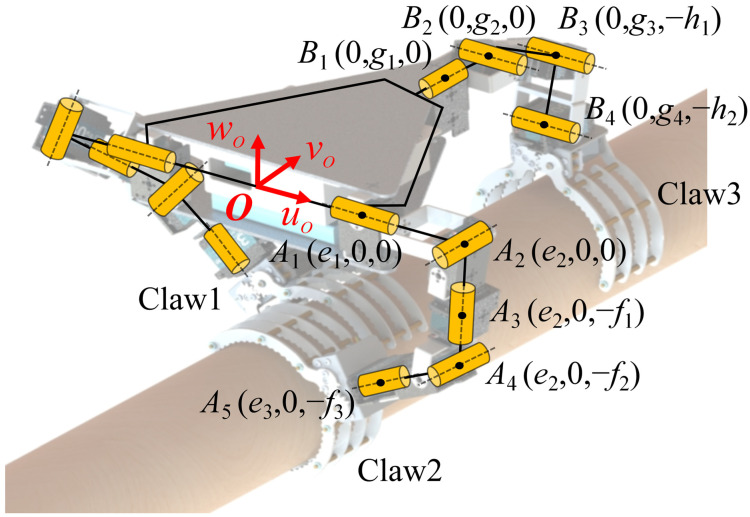
Movement analysis based on screw theory.

The designed three-clawed tree-climbing robot is equipped with servos at all joints, which means that the mobility of the entire mechanism is equal to the number of joints. When the robot needs to ascend or descend, it clamps the tree trunk with the two claws and uses its body as a mobile platform for climbing actions similar to gibbons. To determine the mobility of the robotic body, the relative DoF of the body platform is analyzed using screw theory. Since the inverse and linear correlation of screws are independent of the choice of coordinate system [40], the point *O* on the central axis of the triangular rigid body is selected as the origin of the coordinate system. The positions of the various screw vectors can then be determined, as shown in Figure 3. Taking the manipulator arm where Claw2 is located as an example, its motion screw system is as follows:

(1) $$\begin{array}{c} \begin{array}{lll} {\$}_{2-1} & = & (1\;0\;0;\;0\;0\;0),\\ {\$}_{2-2} & = & (0\;1\;0;\;0\;0\;e_{2}),\\ {\$}_{2-3} & = & (0\;0\;1;\;0-e_{2}\;0),\\ {\$}_{2-4} & = & (0\;1\;0;\;f_{2}\;0\;e_{2}),\\ {\$}_{2-5} & = & (\frac{e_{3}-e_{2}}{\sqrt{{\left(e_{3}-e_{2}\right)}^{2}+{\left(f_{2}-f_{3}\right)}^{2}}}\;0\;\frac{f_{2}-f_{3}}{\sqrt{{\left(e_{3}-e_{2}\right)}^{2}+{\left(f_{2}-f_{3}\right)}^{2}}};\;0\;e_{2}f_{3}-e_{3}f_{2}\;0). \end{array} \end{array}$$

Similarly, the motion screw system of the rear manipulator arm is configured as follows:

(2) $$\begin{array}{c} \begin{array}{l} {\$}_{3-1}=(0\;1\;0;\;0\;0\;0),\\ {\$}_{3-2}=(1\;0\;0;\;0\;0\;-g_{2}),\\ {\$}_{3-3}=(1\;0\;0;\;0\;0\;-h_{1}\;-g_{3}),\\ {\$}_{3-4}=(1\;0\;0;\;0\;0\;-h_{2}\;-g_{4}). \end{array} \end{array}$$

When Claw2 and Claw3 simultaneously grip the tree trunk, combining Equations (1) and (2), the constraint screws acting for the mobile platform are as follows:

(3) $$\begin{array}{c} \begin{array}{cc} {\$}_{3-1}^{r} & =(1\;0\;0;\;0\;0\;0),\\ {\$}_{3-2}^{r} & =(0\;0\;0;\;0\;0\;1),\\ {\$}_{2}^{r} & =(0\;0\;1;\;0\;-e_{2}\;0). \end{array} \end{array}$$

This constraint screw system comprises three spiral constraints, restricting the motion of the mobile platform along the *w_O_* and *u_O_* axes and its rotation around the *w_O_* axis. The body currently has 3 DoFs, yet through coordinated action with the robotic arm, Claw1 achieves a broader range of motion. The rear robotic claw gripping capability significantly enhances stability during grasping in complex environments and can provide reliable support in specific climbing scenarios such as traversing branches.

When Claw1 and Claw2 grip the tree trunk simultaneously, the robotic body can be regarded as a 25R parallel motion platform, where “2” indicates two serial support chains and “5R” means that each chain has five rotational DoFs. The constraint screw for the mobile platform is provided by Equation (4). This screw system forms a pair of force couples, restricting the motion of the mobile platform along the *w_O_*-axis and its rotation around the *u_O_*-axis. At this point, the body has 4 DoFs, allowing it to move along the *u_O_* and *v_O_* axes while simultaneously rotating around the *v_O_* and *w_O_* axes and maintaining the distance between the body and the tree trunk. This method provides the robot with greater freedom of movement, enabling it to climb trees with greater agility and fluidity.

(4) $$\begin{array}{c} \begin{array}{l} {\$}_{1}^{r}=(0\;0\;1;\;0\;e_{2}\;0),\\ {\$}_{2}^{r}=(0\;0\;1;\;0-e_{2}\;0). \end{array} \end{array}$$

### 2.2. Kinematic Modeling of the Bionic Mechanism

According to the DoF analysis, the robot as a hybrid system with three sets of serial manipulator arms has two main operating states: (1) the movement of a single manipulator arm while the other parts of the robot remain stationary; (2) two mechanical claws grip the trunk while the robotic body functions as a parallel motion platform. To enable the robot to achieve the climbing movements of gibbons, this study establishes a kinematic model for the robot during hybrid series–parallel motion. This model not only needs to analyze the motion trajectories of each joint during free swing but also needs to ensure that when the robot grips the trunk with both manipulator arms, its structure can remain stable under a certain load.

To analyze the kinematic issues of the proposed robot structure, the Denavit–Hartenberg (D-H) convention algorithm is used to describe the kinematic relationships of the robot. However, the kinematic solutions in different states are complex, particularly for parallel mechanisms with multiple DoFs and intricate geometric relationships. Direct analytical methods become challenging due to the potential nonlinear constraints between joints. Therefore, this study employs numerical methods to address the kinematic issues of the mechanism.

#### 2.2.1. Single Manipulator Arm Grasping State

**Figure 4:**
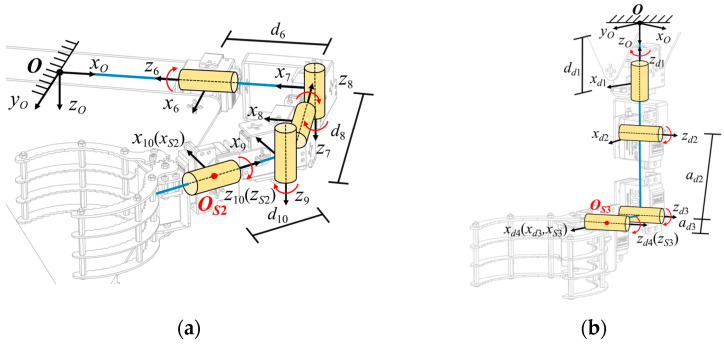
Kinematic model of serial manipulator arm: (**a**) kinematic model of 5R manipulator arm; (**b**) kinematic model of 4R manipulator arm.

Taking the 5R manipulator arm as an example, when a single arm of the robot detaches from the tree trunk to find the next gripping point, the forward and inverse kinematic equations of the end effector of the mechanical claw are based on the body coordinate system. As shown in Figure 4a, the forward coordinate system method is adopted to construct the kinematic model of the 5R manipulator arm.

The D-H parameters for the 5R manipulator arm are listed in Table 1. Then, the general transformation matrix between two joint coordinate frames can be described by Equation (5) [38]:

(5) $$\begin{array}{c} T_{(i-1)-i}=\left[\begin{array}{cccc} \cos\theta_{i} & -\sin\theta_{i} & 0 & \alpha_{i-1}\\ \cos\alpha_{i-1}\sin\theta_{i} & \cos\alpha_{i-1}\cos\theta_{i} & -\sin\alpha_{i-1} & -d_{i}\sin\alpha_{i-1}\\ \sin\alpha_{i-1}\sin\theta_{i} & \sin\alpha_{i-1}\cos\theta_{i} & \cos\alpha_{i-1} & d_{i}\cos\alpha_{i-1}\\ 0 & 0 & 0 & 1 \end{array}\right] \end{array}$$

In conjunction with Table 1, the forward kinematic model of the 5R manipulator arm can be obtained as follows:

(6) $$\begin{array}{ll} T_{6-10} & =T_{06}T_{67}T_{78}T_{89}T_{910}\\ & =\left[\begin{array}{cccc} n_{x2} & o_{x2} & a_{x2} & p_{x2}\\ n_{y2} & o_{y2} & a_{y2} & p_{y2}\\ n_{z2} & o_{z2} & a_{z2} & p_{z2}\\ 0 & 0 & 0 & 1 \end{array}\right] \end{array}$$

The inverse kinematics solution involves determining the corresponding joint angles based on the desired position and orientation coordinate frame of Claw2, which is crucial for controlling the manipulator arm. To solve this problem, the inverse kinematic solution for the 5R manipulator arm is obtained by inverse transformation of the joint transformation matrix. Please refer to Appendix A for a detailed derivation of the closed-form solution for joint angles. Similarly, the rear manipulator arm can use the forward coordinate system method to create a kinematic model of the 4R serial link mechanism and find its kinematic solution, as shown in Equations (A4) and (A5). In addition, the D-H parameters for links 1–5 are listed in Table A3.

#### 2.2.2. Parallel Platform Motion State

**Figure 5:**
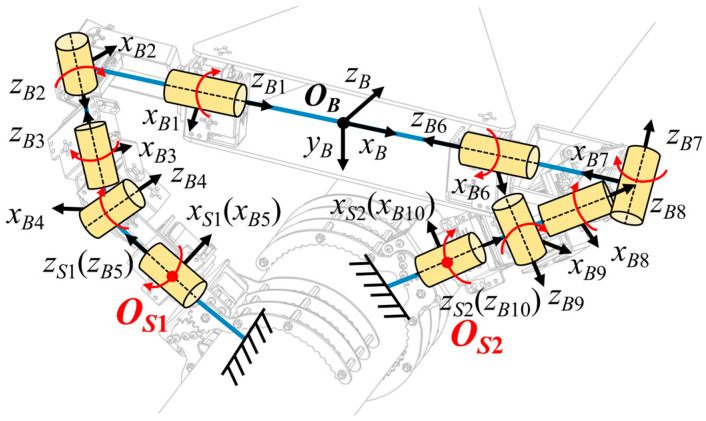
Kinematic model of 2-5R parallel platform.

When Claw1 and Claw2 clamp the tree trunk and remain immobile, the robotic body is regarded as a 2-5R parallel motion platform, as illustrated in Figure 5. In this configuration, *O_S_*_1_ and *O_S_*_2_ are fixed points connected to the tree trunk. The point *O_B_* serves as the control point for the robotic body coordinate frame, with *O_B-xByBzB_* defined as the end-effector coordinate frame. The D-H parameters for the 2-5R parallel platform are detailed in Table A2.

Then, the transformation matrix between the fixed points *O_S_*_1_ and *O_S_*_2_ on the claws and the control point *O_B_* of the robotic body can be established. The frame transformation relationship from the fixed point *O_S_*_1_ on Claw1 to the control point *O_B_* can be determined, which allows the forward kinematic model of the robotic body to be derived:

(7) $$\begin{array}{ll} T_{S1-OB} & =T_{B5-1}T_{1OB}\\ & =\left[\begin{array}{cccc} n_{Ox} & o_{Ox} & a_{Ox} & p_{Ox}\\ n_{Oy} & o_{Oy} & a_{Oy} & p_{Oy}\\ n_{Oz} & o_{Oz} & a_{Oz} & p_{Oz}\\ 0 & 0 & 0 & 1 \end{array}\right] \end{array}$$

(8) $$\begin{array}{ll} T_{S1-OB} & =T_{S1-S2}T_{B10-6}T_{6OB}\\ & =\left[\begin{array}{cccc} n_{Ox} & o_{Ox} & a_{Ox} & p_{Ox}\\ n_{Oy} & o_{Oy} & a_{Oy} & p_{Oy}\\ n_{Oz} & o_{Oz} & a_{Oz} & p_{Oz}\\ 0 & 0 & 0 & 1 \end{array}\right] \end{array}$$

Furthermore, by applying inverse coordinate transformations to the end-effector gripping point and referencing the inverse kinematics solution process, the mapping relationship between the spatial posture adjustment parameters of the robotic 2-5R platform and all joint angles can be obtained. Detailed results provided in Equation (A8).

### 2.3. Design of Numerical Simulation for Model Validation

**Figure 6:**
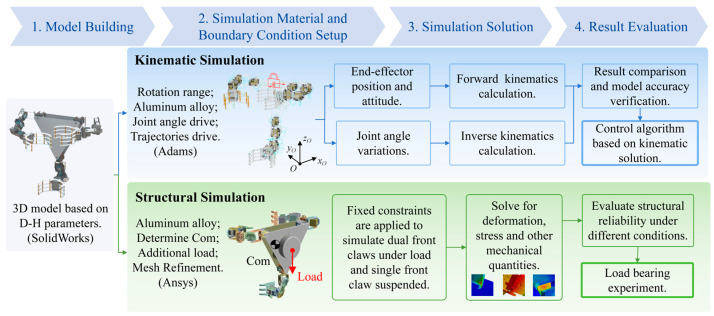
Numerical simulation workflow of the gibbon-inspired tree-climbing robot.

To systematically verify the proposed robotic kinematic model, provide reliable theoretical support for the development of control algorithms, and evaluate the structural reliability of the prototype, two types of numerical simulation experiments were designed in this study. The corresponding flow chart is shown in Figure 6. First, kinematic simulations were carried out based on the Adams View 2017 platform to verify the accuracy of the kinematic models for the serial manipulator arms and the 2-5R parallel moving platform. By presetting the motion trajectories of the end effector, data on the position and attitude changes of the end claw of the manipulator arm and the robot moving platform, as well as the angle variation information of the corresponding rotational joints, were obtained. These two sets of data were then substituted into the kinematic solution formulas to calculate the forward and inverse kinematic results based on the theoretical model. The reliability of the established model was evaluated by comparing these results with the simulation outcomes.

Inspired by the biological characteristic that the skeletal and muscular systems of gibbons can withstand high-load moments during tree climbing, it is necessary to verify whether the designed three-armed robotic structure possesses reliable load-bearing capacity. Therefore, structural simulations were conducted using the ANSYS 2024 R2 finite element simulation software to verify the structural stiffness and anti-overturning capability of the entire robot and its key load-bearing components under loaded conditions so as to ensure the structural stability and operational safety of the physical prototype during actual climbing operations. After importing the established 3D model of the gibbon-inspired tree-climbing robot into ANSYS Workbench 2024 R2, the material properties of the robot model were set to aluminum alloy to determine the center of mass of each component. Following the application of an additional load equivalent to 80% of the robot’s own weight, structural reliability analyses were performed on two typical working conditions during climbing: the working condition with both front claws under load and the working condition with a single front claw suspended in mid-air.

The 3D robot models used in the two numerical simulation experiments were all modeled in SolidWorks 2019 based on the geometric dimensions derived from the D-H parameter table established in Section 2.2. After being imported into the corresponding simulation platforms, material properties, boundary constraints, and load conditions consistent with actual working conditions were strictly configured to minimize the discrepancy between the simulation results and the actual performance of the physical prototype and to guarantee the reliability of the simulations.

## 3. Results

### 3.1. Validation of Kinematic Modeling

**Figure 7:**
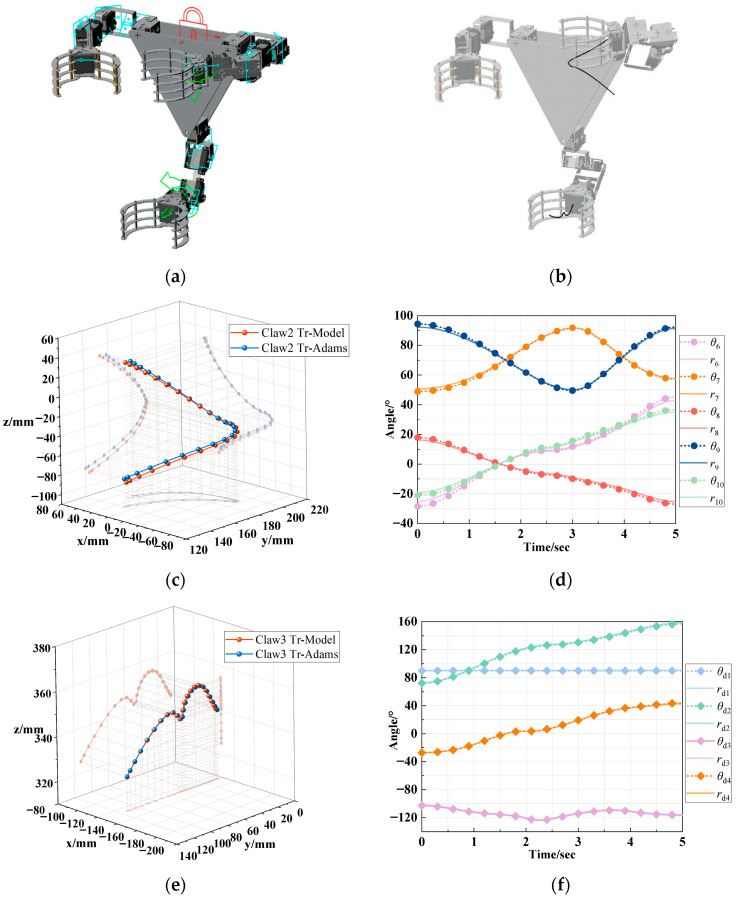
Numerical simulation of kinematic models for serial manipulator arms: (**a**) simulation environment setting; (**b**) trajectories of Claw2 and Claw3; (**c**) forward kinematic model validation of 5R manipulator arm; (**d**) inverse kinematic model validation of 5R manipulator arm; (**e**) forward kinematic model validation of 4R manipulator arm; (**f**) inverse kinematic model validation of 4R manipulator arm.

When the forward kinematic model of the serial manipulator arm was verified, the robotic body was fixed to the ground, and a motion was added at the center of mass of Claw2, as shown in Figure 7a. After setting the simulation time, Claw2 was used as the end effector to perform the motion, and the resulting trajectory curve is shown in Figure 7b. Upon completion of the simulation, the movement curve of the center of mass of Claw2 and the rotation curves of each joint on the arm were exported. The motion trajectories are plotted and compared with the simulated trajectories of the Claw2 center of mass, using the forward kinematic model in Equation (A1), with the joint rotation data entered to calculate the end-effector coordinates for each sample point. Figure 7c shows that the two trajectories are essentially the same. Analysis of the root mean square error (RMSE) and maximum deviation (Table 2) showed an RMSE of 3.86 mm and a maximum deviation of 6.76 mm, which is 2.95% of the range of motion. The error is mainly due to positional differences between the joint motion coordinate frame in the model and the center of mass frame established by Adams View 2017. However, this has little to no impact on the motion of the tree-climbing robot. Therefore, the established forward kinematic model of the 5R manipulator arm is considered valid.

To validate the inverse kinematic model of the serial manipulator arm, using the inverse kinematic solution obtained in Equation (A3), the joint rotation angles for each sample point were calculated and plotted. This data is then compared with the simulated joint rotation curves from Adams View 2017 for the arm of Claw2, as shown in Figure 7d. The maximum variation in joint angles was observed for rod 6, with an RMSE of 1.69°and a maximum deviation of 3.21°, representing 4.72% of its rotation range. These error values are considered acceptable for the motion control of the tree-climbing robot. This result confirms the validity of the inverse kinematic model of the 5R manipulator arm. Similarly, a motion was added to Claw3, and simulations were carried out. The results were compared with the outputs of the 4R manipulator arm kinematic model obtained in Equations (A4) and (A5). The comparison results shown in Figure 7e,f also validate the effectiveness of the developed model.

**Figure 8:**
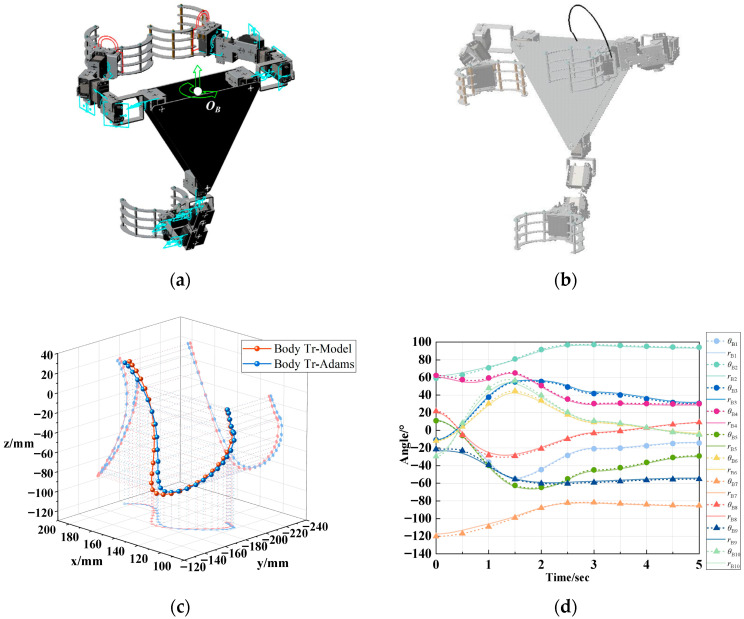
Numerical simulation of kinematic model for 2-5R parallel platform: (**a**) simulation environment setting; (**b**) trajectory of *O_B_*; (**c**) forward kinematic model validation of 2-5R parallel platform; (**d**) inverse kinematic model validation of 2-5R parallel platform.

To validate the kinematic model of the 2-5R parallel platform, the simulation conditions were reset: the fixed ground and the body were deactivated, and Claw1 and Claw2 were fixed to simulate the clamping of the tree trunk. A motion was added to the body, and after simulation, the trajectory curve of the drive point *O_B_* was obtained, as shown in Figure 8b. The rotation curves of all joints on the manipulator arms of Claw1 and Claw2 were measured and the relevant data exported. Figure 8c,d show that the joint rotation curves and the overall movement trajectory of the robot closely match the results of the model. The deviation analysis shown in Table 3 reveals that for the forward kinematic model, the RMSE of the platform trajectory is 3.69 mm with a maximum deviation of 6.07 mm, representing 2.41% of its range of motion. For the inverse kinematic model, the maximum deviation in joint angles is observed for rod 10, with an RMSE of 2.31° and a maximum deviation of 5.29°, representing 13.68% of its range of motion. The model deviation was larger compared to the verification of the serial model due to the increased computational complexity and the accumulation of measurement and positioning errors. However, this margin of error is acceptable for clamping on rough bark surfaces, and the data trends align with the simulation results. Consequently, the 2-5R parallel platform kinematic model is fully validated as an effective solution for the control of robots, providing accurate theoretical support.

### 3.2. Validation of Structural Stiffness

**Figure 9:**
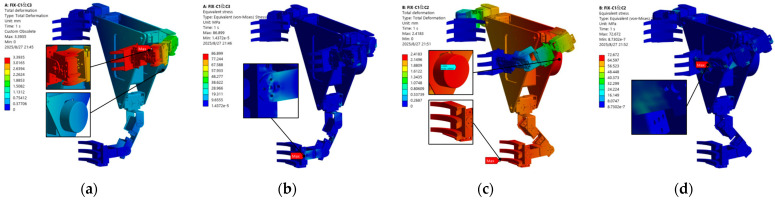
Structural performance analysis of the robot: (**a**) single front claw suspended—total deformation distribution contour plot; (**b**) single front claw suspended—equivalent stress distribution contour plot; (**c**) both front claws loaded—total deformation distribution contour plot; (**d**) both front claws loaded—equivalent stress distribution contour plot.

Based on the structural simulation experiments designed in Section 2.3, this section validates the structural stiffness and anti-overturning capability of the robot model under typical load conditions. The simulation results are presented in Figure 9. The total deformation contour plot indicates that under load conditions, the maximum deformation occurs at the extremity of the robotic arm when a single forelimb is suspended, amounting to approximately 3.4 mm. Core load-bearing components, such as joint fasteners and gripper claws, exhibited deformations below 1.8 mm. This demonstrates the capacity of the robot structure to resist overturning moments induced by the load, with small displacement arising from structural deformation. The equivalent stress contour plot shows a maximum stress of 86.9 MPa, which is below the allowable stress limit of 240 MPa for 6061 aluminum alloy. Furthermore, no significant stress concentrations were observed, thereby validating the structural reliability of the robot. When Claw1 and Claw2 clamped simultaneously, the maximum total structural deformation was 2.4 mm with a peak stress of 72.6 MPa; both values were below the safety threshold. The aforementioned mechanical simulation not only validated the overall structural rigidity of the robot but also demonstrated the soundness of the simplified design featuring both front claws’ cooperative load-bearing coupled with single-hindlimb auxiliary thrust. This enables the robot to effectively resist overturning moments imposed by external forces, while incurring minimal structural deformation.

### 3.3. Control System and Prototype Fabrication Based on Simulation Results

**Figure 10:**
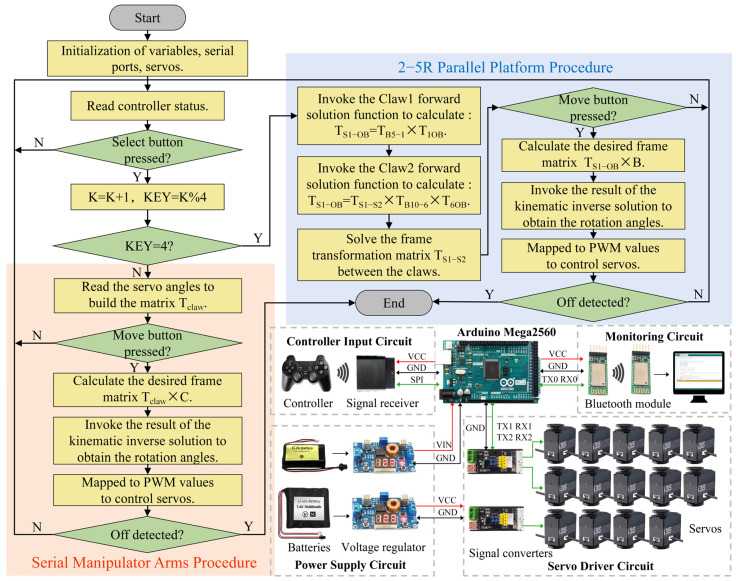
Robot control system and hardware configuration based on kinematic solutions.

Direct control of the position and orientation during robot manipulation is a more user-friendly solution. Therefore, based on the kinematic solutions obtained in Section 2.2, a control program is developed. The operator uses the *Select* button on the controller to change the value of the variable *KEY*, which switches the control target to either the claws or the robotic platform, providing intuitive control. When the value of *KEY* is not equal to 4, the control is executed using the claw as the end effector. The serial control procedure for the manipulator arms is invoked using the joint servo angles as input. Forward kinematic is used to obtain the current coordinate frame matrix *T_claw_* of the claw control point. The operator adjusts the matrix operator *C* through move commands, giving the desired coordinate frame matrix *T_claw_* × *C*. The required joint angles are calculated using the inverse kinematic results from Section 2.2. These angles are mapped to PWM (pulse width modulation) values and sent to the joint servos to move the claw to the desired coordinate frame. When *KEY* = 4, the robot enters the 2-5R platform control mode, and the parallel procedure is invoked. The control program flowchart is shown in Figure 10.

The above control flow is compiled into a program and written to the main controller board through the Arduino IDE. Furthermore, the program incorporates multiple action sequences to enable quick adjustments to the posture of the manipulator arm or the robotic body. The hardware configuration of the control system includes the following: Arduino Mega2560 is chosen as the main control board for processing commands and transmitting motion signals; a controller input circuit for wireless transmission of commands; a monitoring circuit based on a Bluetooth module for communication with the host computer and visualization of operations; a servo driver circuit that transmits motion commands to the servo group and returns angle information; a power supply circuit with lithium batteries and voltage regulator board to provide stable voltage to the main controller board and servo drivers.

In order to verify the kinematic performance of the designed tree-climbing robot system, a physical prototype was fabricated based on the above control strategies, and field climbing experiments were conducted. The main structural parts of the robot are made of aluminum alloy to satisfy the demands for structural strength and lightweight design. Some non-load-bearing components are fabricated through 3D printing with photosensitive resin, which not only effectively reduces the overall weight but also lowers the processing cost of parts. The total mass of the final prototype is approximately 2.52 kg. After wiring layout and the installation of the power fixing plate, core hardware, including lithium batteries and control boards, is integrated into the robot robotic body. The robotic body has a length of 236 mm, a width of 202 mm, and a height of 74 mm, with a weight of 1.36 kg, accounting for about 54% of the robot’s total weight. Such mass distribution concentrates most of the overall mass in the middle section of the robot, which can effectively guarantee the stability of the center of gravity during climbing.

The clamping claws equipped on the manipulator arms are commercial mass-produced servo mechanical claws. Adopting aluminum alloy as the main material, they can adapt to trunk gripping with diameters ranging from 50 to 135 mm. The robot operates by receiving PWM signals that drive the servo motor’s rotor, turning the gear structure to control the opening and closing of claw fingers and realize stable trunk clamping. A single mechanical claw weighs merely 190 g with the servo installed, greatly reducing the overall load of the robot. The claw fingers are designed with a serrated structure to provide anti-slip capability, which enhances the gripping reliability on tree trunks. The front manipulator arms are equipped with five rotating servo motors, with a total weight of about 407 g. Through actual assembly, debugging, and dimensional measurement, the maximum extended length of the front arms reaches approximately 393 mm. The rear manipulator arms feature a compact structure, weighing 376 g in total and reaching a maximum extended length of 328 mm. Measured in the preliminary debugging stage, the overall external dimension of the robot under working conditions is 590 mm in length, 710 mm in width, and 340 mm in height.

### 3.4. Grasping and Load-Bearing Stability Experiments

**Figure 11:**
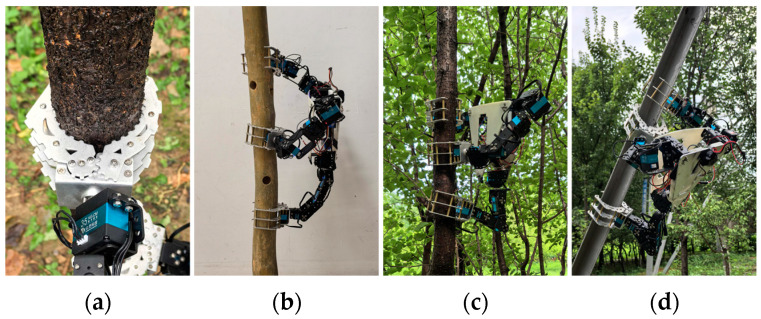
Robotic claw grasping experiments: (**a**) metal mechanical claw; (**b**) grasping smooth artificial wood; (**c**) grasping a damp tree trunk after rainfall; (**d**) grasping steel truss.

To evaluate the performance of the claw, robotic grasping tests were conducted on surfaces made of different materials. As illustrated in Figure 11, the mechanical claw is capable of achieving stable clamping on surfaces with varying roughness, including moist tree trunk, artificial wood blocks, and smooth carbon steel sheets. The clamped experimental materials differ greatly in hardness and surface smoothness, with their specific parameters presented in Table 4.

**Figure 12:**
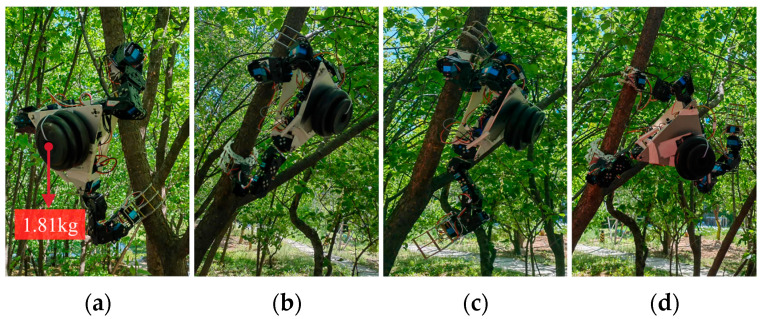
Robot load capacity experiments: (**a**) maximum counterweight of 1.81 kg; (**b**) three claws clamping; (**c**) two front claws clamping; (**d**) one front and one rear claw clamping.

Furthermore, a load-bearing stability experiment was carried out to test the stability of the three-arm clawed robot under external loads. Based on the opening and closing ranges of the claws, plum tree trunks with diameters of 72 mm and an inclination angle of approximately 70° with the ground were selected as gripping objects in order to simulate the typical conditions that the robot might encounter when gripping. The load-bearing capacity of the prototype was verified by gradually adding counterweights to the central rigid body. The performance of the robot under different gripping conditions was then observed to determine whether failure would occur, as shown in Figure 12. The results showed that the robot remained stable when gripping with three claws and simultaneously gripping with the front claws. However, in the case of one front and one rear claw clamp, the robot exhibited a significant tilt due to an excessive shift in the center of gravity, making it prone to tipping over. Under the condition of ensuring gripping stability, the robot was able to bear a maximum counterweight of approximately 1.81 kg, which is 71.8% of its own weight.

### 3.5. Robot Tree-Climbing Capability Validation Experiments

**Figure 13:**
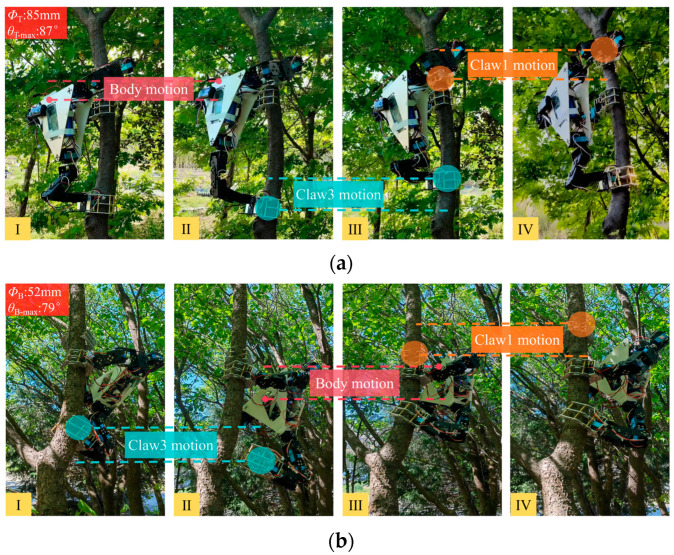
Robot tree-climbing capability validation experiments: (**a**) robot dynamic climbing on the trunk; (**b**) robot dynamic climbing on the branch.

Dynamic climbing experiments were also conducted to further evaluate the practical performance of the tree-climbing robot. In the experiment, a near-vertical plum tree trunk with a diameter of 85 mm was selected as the climbing object to simulate common climbing scenarios. Following the control logic based on the kinematic solution, the robot was manipulated by the joystick controller and successfully completed a series of climbing actions. The input action commands included adjusting the opening and closing of the claws, overall ascending of the platform, performing actions with each of the robot manipulator arms, etc. Figure 13a shows the sequence of a complete upward climbing gait. To further verify the adaptability of the robot to non-vertical branches, a branch of a plum tree with a diameter of 52 mm and an inclination angle of 79° was selected as the climbing target, and a complete upward climbing gait was successfully completed, as shown in Figure 13b. The result demonstrated that the robot is capable of climbing on relatively thin branches. However, due to the limited clamping range of the claws, it is unable to grasp branches with extremely small diameters.

**Figure 14:**
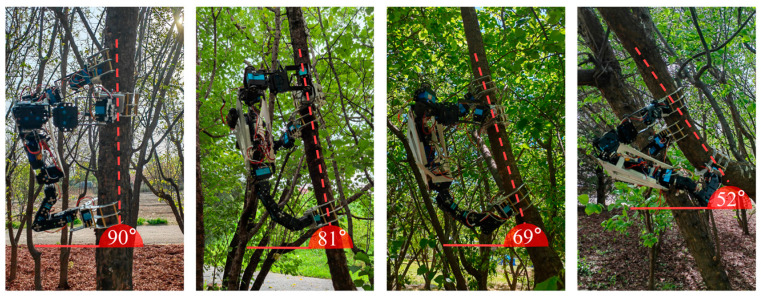
Climbing tests on tree trunks with different inclination angles.

In addition, multiple sets of field climbing tests were carried out under forest conditions with different trunk diameters and inclination degrees. Based on the statistical results of 16 valid and complete climbing gaits, the robot could stably respond to control commands in various field environments. The average duration of a single complete climbing gait was approximately 4 min, with a fluctuation range of 2.6–6.1 min. The time variation was mainly caused by the irregular surface features of natural tree trunks, and repeated fine adjustments of gripping positions were required when clamping on trunks with a large inclination angle. Furthermore, the single lifting distance of the robot body ranged from 17 cm to 24 cm. The maximum effective movement distance of the front manipulator claw reached 36 cm, while the maximum ascending distance of the rear manipulator claw was 27 cm. Verified by practical tests, the robot can steadily climb inclined trunks with a ground inclination angle ranging from 52° to 90°, as shown in Figure 14, which enables it to adapt to common forest working scenarios.

## 4. Discussion

This study systematically verifies the gibbon-inspired tree-climbing robot through kinematic modeling, structural simulation, and field prototype experiments. Climbing experiments showed that the series–parallel control scheme based on kinematic solutions was able to effectively direct the robot to complete stable climbing movements, which further validates its operational performance in dynamic field environments. The prototype has a total mass of approximately 2.52 kg and a maximum total length of 0.59 m. Compared with existing small-scale climbing robots, such as the 6.8 kg tree-climbing robot [41] and the 5.4 kg hook-claw robot “RISE V3” [42], this design achieves lightweight performance, which effectively reduces the overall weight and simplifies the control system. In contrast to the 42 kg “Climbot” [25,26] with a total length of about 2.5 m, which is specially designed for rod climbing, the proposed robot can pass through narrow branches and delivers better environmental flexibility.

Wheeled pole-climbing robots can achieve a high moving speed of 80 cm/s [43]. By comparison, the claw-type tree-climbing robot proposed in this study requires approximately 4 min to finish a single gait cycle, with a climbing speed inferior to the 5.75 cm/s of the similarly sized “PCRobot” [24]. This limitation mainly stems from redundant design in the control program and response delay between instruction analysis and motion execution, which impairs the continuity of climbing movements during the operation of serial manipulators and the parallel moving platform. In addition, owing to the irregular surface features of natural tree trunks, multiple adjustments of gripping positions are required during clamping to ensure stable attachment, which further prolongs the operating time of each climbing gait.

Despite the disadvantage of relatively slow climbing speed, this bionic design presents remarkable strengths in terms of operating space and load-bearing capacity. Specifically, the robot can maintain a distance of 80–200 mm from the tree trunk, while traditional hook-claw robots such as the “Treebot” [22,23] and the hexapod robot developed by Spenko et al. [27] need to fit closely to the trunk. This distinctive feature enables the gibbon-inspired robot to cross obstacles and climb on trunks with an inclination of 52–90°, avoiding bark damage caused by direct body friction. Meanwhile, benefiting from additional gripping points, the three-clawed structure greatly improves the load-bearing capacity in actual climbing scenarios, with a maximum payload of 1.81 kg and a load-to-weight ratio of approximately 72%. Its bearing capacity is about nine times higher than that of the bionic spine climbing robot designed by Liu et al. [44].

It was found during dynamic climbing experiments that when a single claw loses power, the robot can still adhere to the trunk by virtue of gravity due to the spatial positional relationship among claws. Further analysis and structural optimization are expected to enhance the environmental adaptability and mechanical durability of the robot. Experimental observations also indicated that excessive start-up acceleration of servo motors and low mechanical structural stiffness easily cause obvious sway during the large-range movement of a single front manipulator arm, which may adversely affect climbing stability and safety. This swaying issue can be effectively alleviated by adopting polynomial interpolation for trajectory planning. Concurrently, a model of adjustable manipulator arm stiffness would be developed to enable compliant motion by adjusting joint stiffness in real time [45].

## 5. Conclusions

This paper presents a three-armed clawed tree-climbing robot; through the bionic analysis of the structural characteristics of gibbons and their climbing action, a robot model with 14 total DoFs and 4 body motion DoFs was constructed. Compared to conventional designs, the climbing robot proposed in this study has a distinct structural layout and motion pattern. Its hybrid serial–parallel structure eliminates redundant joints, ensuring stability and significantly simplifying the control system. To verify the rationality of the three-manipulator structural design, systematic numerical simulation analysis was conducted in this study: Kinematic simulations were performed to verify the accuracy of the series manipulator arm and parallel platform models. Finite element analysis was employed to simulate gripping scenarios during tree-climbing operations, and the mechanical properties of the robot’s core components under loaded conditions were obtained: the maximum deformation was only 2.4 mm, and the peak stress reached merely 30.3% of the material’s yield strength, which is far below the safety threshold. Combined with the fact that no obvious deformation or wear was observed on the key components of the prototype after the experiment, support for the safety of the robot’s structural strength was provided from both numerical simulation and physical verification perspectives. These research results informed the design of a robot control system using kinematic solutions and supporting hardware configurations, and a prototype was developed.

The experimental results indicate that the claws can provide stable gripping force on surfaces of materials with different roughness, such as moist tree bark and smooth carbon steel sheets. Meanwhile, the robot has a certain load-bearing capacity, with a maximum load-bearing capacity of 1.81 kg. In actual climbing experiments, the robot successfully completed various operational actions, effectively simulating the climbing scenario of a single tree, which demonstrates the reliability and efficiency of the robot system in practical applications. Dynamic climbing experiments further verified its environmental adaptability: the robot is capable of climbing branches and tree trunks with a diameter of 50–135 mm and an inclination angle greater than 52°. It is evident that the range of the mechanical claw is directly proportional to the dimensions of the claw, with the capacity to extend the working space through the replacement of either the claws or associated modules. In conclusion, based on existing tree-climbing robot technologies, this study has completed the design and prototype development of a gibbon-inspired clawed tree-climbing robot system. The proposed robot adopts a hybrid series–parallel configuration with three manipulator arms, providing a feasible robotic solution for safe high-altitude operations in forest areas.

Nevertheless, this research still has certain limitations. Restricted by the control system and cumbersome clamping motions, the operational efficiency of the robot remains to be further improved. Current experiments were only conducted on natural tree trunks with limited inclination angles and diameters. Under controlled variable conditions, the influence of trunk curvature on the climbing performance of the robot has not been investigated, nor has the motion accuracy of the claws and manipulator arms been explored. Furthermore, only basic climbing and load-bearing functions are realized at present, while practical forestry operation modules have not been integrated, leading to restricted test scenarios. Therefore, future work will build a controllable indoor experimental platform to quantitatively analyze various factors affecting the climbing performance of the robot system. By simplifying the operation logic, optimizing redundancies in the control program, and combining polynomial interpolation to realize compliant motion control, the climbing efficiency and manipulation accuracy of the robot will be improved. A universal modular structure will also be designed to facilitate the installation of pruning, fruit picking, sensing, and other operational equipment so as to develop a multifunctional forestry operation platform. These improvements will further expand the application scenarios and operation capabilities of this gibbon-inspired robot, thereby enhancing its practical application performance in complex forestry environments.

## Figures and Tables

**Table 1:** D-H parameters table of 5R manipulator arm.

Rod *i*	*α_i_*_−1_ (°)	*a_i_*_−1_ (mm)	*d_i_* (mm)	*θ_i_* (°)
6	0	0	*d*_6_ = −61	*θ*_6_
7	90	0	0	*θ*_7_
8	−90	0	*d*_8_ = −122	*θ*_8_
9	90	0	0	*θ*_9_
10	−90	0	*d*_10_ = −90	*θ*_10_

Note: *α_i_*_−1_—the link twist angle between joint *i*−1 and *i*; *a_i_*_−1_—the link length between joint *i*−1 and *i*; *d_i_*—the link offset between joint *i*−1 and *i*; *θ_i_*—the rotation angle of joint *i*.

**Table 2:** Serial kinematic model deviation.

	5R Trajectory Deviation (mm)	*θ*_6_ (°)	*θ*_7_ (°)	*θ*_8_ (°)	*θ*_9_ (°)	*θ*_10_ (°)	4R Trajectory Deviation (mm)	*θ_d_*_1_ (°)	*θ_d_*_2_ (°)	*θ_d_*_3_ (°)	*θ_d_*_4_ (°)
RMSE	3.86	1.69	0.80	1.01	1.10	1.29	1.14	0.00	0.79	0.12	0.78
Maximum deviations	6.76	3.21	1.48	1.97	2.31	1.94	1.84	0.00	1.35	0.27	1.49

**Table 3:** Kinematic model deviation of 2-5R parallel platform.

	Parallel Platform Trajectory Deviation (mm)	*θ_B_*_1_ (°)	*θ_B_*_2_ (°)	*θ_B_*_3_ (°)	*θ_B_*_4_ (°)	*θ_b_*_5_ (°)	*θ_B_*_6_ (°)	*θ_B_*_7_ (°)	*θ_B_*_8_ (°)	*θ_B_*_9_ (°)	*θ_B_*_10_ (°)
RMSE	3.69	0.22	1.42	1.06	1.54	1.35	1.33	1.63	1.45	1.42	2.31
Maximum deviations	6.07	0.28	2.43	1.47	2.39	1.67	2.77	3.53	3.85	3.28	5.29

**Table 4:** Parameter table of materials used in grasping experiments.

Test Samples		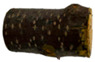 Purple-Leaf Plum	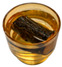 Purple-Leaf Plum (Moist)	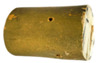 Artificial Wood	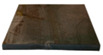 Q235B
Surface roughness	Ra/μm	9.88	10.92	10.09	1.53
	Rz/μm	63.42	49.10	53.90	9.79
Hardness		D:42.0	D:33.5	D:55.3	HRB:72.5
Static friction coefficient with aluminum alloy		0.50	0.55	0.41	0.31

## Data Availability

Data is contained within the article.

## Appendix A

Combining the D-H parameters in Table 1 with the complete forward kinematic model of the 5R manipulator arm (Equation (6)), detailed results are provided in the following expression:

(A1) $$\begin{array}{c} \begin{array}{ll} n_{x2} & =-C_{10}\left[C_{9}(S_{6}S_{8}-C_{6}C_{7}C_{8})+C_{6}S_{7}S_{9}\right]-S_{10}\left(S_{6}C_{8}+C_{6}C_{7}S_{8}\right)\\ n_{y2} & =C_{10}\left[C_{9}\left(C_{6}S_{8}+C_{7}C_{8}S_{6}\right)-S_{6}S_{7}S_{9}\right]+S_{10}\left(C_{6}C_{8}-S_{6}C_{7}S_{8}\right)\\ n_{z2} & =C_{10}\left(C_{7}S_{9}+S_{7}C_{8}C_{9}\right)-S_{7}S_{8}S_{10}\\ o_{x2} & =-C_{10}\left(S_{6}C_{8}+C_{6}C_{7}S_{8}\right)+S_{10}\left[C_{9}\left(S_{6}S_{8}-C_{6}C_{7}C_{8}\right)+C_{6}S_{7}S_{9}\right]\\ o_{y2} & =C_{10}\left(C_{6}C_{8}-S_{6}C_{7}S_{8}\right)-S_{10}\left[C_{9}\left(C_{6}S_{8}+S_{6}C_{7}C_{8}\right)-S_{6}S_{7}S_{9}\right]\\ o_{z2} & =-S_{10}\left(C_{7}S_{9}+S_{7}C_{8}C_{9}\right)-S_{7}S_{8}C_{10}\\ a_{x2} & =S_{9}\left(S_{6}S_{8}-C_{6}C_{7}C_{8}\right)-C_{6}S_{7}C_{9}\\ a_{y2} & =-S_{9}\left(C_{6}S_{8}+S_{6}C_{7}C_{8}\right)-S_{6}S_{7}C_{9}\\ a_{z2} & =C_{7}C_{9}-S_{7}C_{8}S_{9}\\ p_{x2} & =-d_{8}C_{6}S_{7}+d_{10}\left[S_{9}\left(S_{6}S_{8}-C_{6}C_{7}C_{8}\right)-C_{6}S_{7}C_{9}\right]\\ p_{y2} & =-d_{8}S_{6}S_{7}-d_{10}\left[S_{9}\left(C_{6}S_{8}+S_{6}C_{7}C_{8}\right)+S_{6}S_{7}C_{9}\right]\\ p_{z2} & =d_{6}+d_{8}C_{7}+d_{10}\left(C_{7}C_{9}-S_{7}C_{8}S_{9}\right) \end{array} \end{array}$$

To simplify the expression, define *S_i_* as sin (*θ_i_*) and *C_i_* as cos (*θ_i_*). Multiplying by the inverse matrix of the left-hand side of Equation (6) progressively feeds back the position and orientation information of Claw 2 to each joint, as shown in Equation (A2).

(A2) $$\begin{array}{c} {T_{06}}^{-1}T_{6-10}=T_{67}T_{78}T_{89}T_{910} \end{array}$$

Since the elements of the left and right sides of the Equation are equal, it is possible to solve angles of *θ*_6_ and *θ*_7_ first. Similarly, Equation (A3) can be obtained by performing another inverse matrix transformation. These results provide the complete inverse kinematic solution for the 5R manipulator arm, which is important for the control of Claw2.

(A3) $$\begin{array}{c} \begin{array}{ll} \theta_{6} & =\arctan2\left(\frac{p_{y2}-d_{10}a_{y2}}{d_{8}},\frac{p_{x2}-d_{10}a_{x2}}{d_{8}}\right)\\ \theta_{7} & =\arctan2\left(\frac{-p_{x2}C_{6}+p_{y2}S_{6}+d_{10}(a_{x2}C_{6}+a_{y2}S_{6})}{d_{8}},\frac{p_{z2}-d_{6}-d_{10}a_{z2}}{d_{8}}\right)\\ \theta_{8} & =\arctan2\left(\frac{p_{y2}C_{6}-p_{x2}S_{6}}{p_{x2}C_{6}C_{7}+p_{y2}S_{6}C_{7}+p_{z2}S_{7}-d_{6}S_{7}}\right)\\ \theta_{10} & =\arctan2\left(\frac{o_{x2}C_{6}S_{7}+o_{y2}S_{6}S_{7}-o_{z2}C_{7}}{-n_{x2}C_{6}S_{7}-n_{y2}S_{6}S_{7}+n_{z2}C_{7}}\right)\\ \theta_{9} & =\arctan2\left(\frac{n_{z2}C_{7}-n_{x2}C_{6}S_{7}-n_{y2}S_{6}S_{7}}{C_{10}},\frac{a_{z2}C_{7}-a_{x2}C_{6}S_{7}-a_{y2}S_{6}S_{7}}{C_{10}}\right) \end{array} \end{array}$$

Referring to the above process, the forward kinematics solution in Equation (A4) and the inverse kinematics solution in Equation (A5) for the 4R manipulator arm can be obtained.

**Table A1:** D-H parameters table of 4R manipulator arm.

Rod *i*	*α_di_*_−1_ (°)	*a_di_*_−1_ (mm)	*d_di_* (mm)	*θ_di_* (°)
*d*_1_	0	0	*d**_d_*****_1_** = 27	*θ_d_*_1_
*d*_2_	90	0	0	*θ_d_*_2_
*d*_3_	0	*a_d_*_2_ = 83	0	*θ_d_*_3_
*d*_4_	0	*a_d_*_2_ = 55	0	*θ_d_*_4_

(A4)
$$\begin{array}{c} \begin{array}{l} n_{dx}=C_{d1}[C_{d4}(C_{d2}C_{d3}-S_{d2}S_{d3})-S_{d4}(C_{d2}S_{d3}+S_{d2}C_{d3})]\\ n_{dy}=S_{d1}[C_{d4}(C_{d2}C_{d3}-S_{d2}S_{d3})-S_{d4}(C_{d2}S_{d3}+S_{d2}C_{d3})]\\ n_{dz}=C_{d4}(C_{d2}S_{d3}+S_{d2}C_{d3})-S_{d4}(S_{d2}S_{d3}-C_{d2}C_{d3})\\ o_{dx}=-C_{d1}[C_{d4}(C_{d2}S_{d3}+S_{d2}C_{d3})+S_{d4}(C_{d2}C_{d3}-S_{d2}S_{d3})]\\ o_{dy}=-S_{d1}[C_{d4}(C_{d2}S_{d3}+S_{d2}C_{d3})+S_{d4}(C_{d2}C_{d3}-S_{d2}S_{d3})]\\ o_{dz}=-C_{d4}(S_{d2}S_{d3}-C_{d2}C_{d3})-S_{d4}(S_{d2}C_{d3}+C_{d2}S_{d3})\\ a_{dx}=S_{d1}\\ a_{dy}=-C_{d1}\\ a_{dz}=0\\ p_{dx}=a_{d3}C_{d1}(C_{d2}C_{d3}-S_{d2}S_{d3})+a_{d2}C_{d1}C_{d2}\\ p_{dy}=a_{d3}S_{d1}(C_{d2}C_{d3}-S_{d2}S_{d3})+a_{d2}S_{d1}C_{d2}\\ p_{dz}=d_{d1}+a_{d3}(C_{d2}S_{d3}+C_{d3}S_{d2})+a_{d2}S_{d2} \end{array} \end{array}$$

(A5)
$$\begin{array}{c} \begin{array}{ll} \theta_{d1} & =\arctan2(a_{dx},-a_{dy})\\ \theta_{d3} & =-\arctan\left(\frac{(p_{dz}-d_{d1}{)}^{2}+(p_{dx}C_{d1}+p_{dy}S_{d1}{)}^{2}-a_{d2}^{2}-a_{d3}^{2}}{2a_{d2}a_{d3}}\right)\\ \theta_{d2} & =\arctan\left(\frac{\left(a_{d2}+a_{d3}C_{d3})(p_{dz}-d_{d1})-S_{d3}a_{d3}(p_{dx}C_{d1}+p_{dy}S_{d1}\right)}{S_{d3}a_{d3}(p_{dz}-d_{d1})+(a_{d2}+a_{d3}C_{d3})(p_{dx}C_{d1}+p_{dy}S_{d1})}\right)\\ \theta_{d4} & =\arctan2(n_{dz},C_{d1}n_{dx}+S_{d1}n_{dy})-\theta_{d2}-\theta_{d3} \end{array} \end{array}$$

For the 2-5R platform, the D-H parameter table is given in Table A2.

**Table A2:** D-H parameters table of 2-5R parallel platform.

Rod *i*	*α_Bi_*_−1_ (°)	*a_Bi_*_−1_ (mm)	*d_Bi_* (mm)	*θ_Bi_* (°)
10	0	0	*d**_B_*****_10_** = 90	*θ* *_B_*_10_
9	90	0	0	*θ* *_B_*_9_
8	−90	0	*d**_B_*****_8_** = 122	*θ* *_B_*_8_
7	90	0	0	*θ* *_B_*_7_
6	−90	0	*d**_B_*****_6_** = 61	*θ* *_B_*_6_
5	90	0	*d**_B_*****_5_** = 90	*θ* *_B_*_5_
4	−90	0	0	*θ* *_B_*_4_
3	90	0	*d**_B_*****_3_** = 125	*θ* *_B_*_3_
2	−90	0	0	*θ* *_B_*_2_
1	90	0	*d**_B_*****_1_** = 58	*θ* *_B_*_1_

In Equation (8), *T*_6_*_OB_* can be obtained from Figure 5, while *T_S_*_1−_*_S_*_2_ represents the relationship between the two claw points after the serial movement of the manipulator arms. *T_B_*_10−6_ denotes the inverse transformation of rod 10 to rod 6 on the manipulator arm of Claw2. In order to control the movement of the platform, an inverse kinematic analysis of the body is also required. This involves solving the required angle of rotation of the joints, given the desired coordinate frame matrix *T_S_*_1−_*_OB_*, and thus, the expected coordinate frames *T_S_*_1−1_ and *T_S_*_2−6_ for the two manipulator arms are as follows:

(A6) $$\begin{array}{ll} T_{S1-1} & =T_{B5-1}=T_{S1-OB}{T_{1OB}}^{-1}\\ & =\left[\begin{array}{cccc} n_{Bx1} & o_{Bx1} & a_{Bx1} & p_{Bx1}\\ n_{By1} & o_{By1} & a_{By1} & p_{By1}\\ n_{Bz1} & o_{Bz1} & a_{Bz1} & p_{Bz1}\\ 0 & 0 & 0 & 1 \end{array}\right] \end{array}$$

(A7) $$\begin{array}{ll} T_{S2-6} & =T_{B10-6}={T_{S1-S2}}^{-1}T_{S1-OB}{T_{6OB}}^{-1}\\ & =\left[\begin{array}{cccc} n_{Bx2} & o_{Bx2} & a_{Bx2} & p_{Bx2}\\ n_{By2} & o_{By2} & a_{By2} & p_{By2}\\ n_{Bz2} & o_{Bz2} & a_{Bz2} & p_{Bz2}\\ 0 & 0 & 0 & 1 \end{array}\right] \end{array}$$

The mapping relationship between the robotic body coordinate frame and all joint angles can be obtained by associating the above equations, as described in Equation (A8). Thus, the inverse kinematic solution of the 2-5R parallel system is obtained.

(A8) $$\begin{array}{c} \begin{array}{ll} \theta_{B10}= & \arctan2(\frac{p_{By2}-d_{B6}a_{By2}}{d_{B8}},\frac{p_{Bx2}-d_{B6}a_{Bx2}}{d_{B8}})\\ \theta_{B9}= & \arctan2(\frac{-p_{Bx2}C_{B10}-p_{By2}S_{B10}+d_{B6}(a_{Bx2}C_{B10}+a_{By2}S_{B10})}{d_{B8}},\frac{p_{Bz2}-d_{B10}-d_{B6}a_{Bz2}}{d_{B8}})\\ \theta_{B8}= & \arctan(\frac{a_{By2}C_{B10}-a_{Bx2}S_{B10}}{a_{Bx2}C_{B9}C_{B10}+a_{By2}C_{B9}S_{B10}+a_{Bz2}S_{B9}})\\ \theta_{B6}= & \arctan(\frac{o_{Bx2}C_{B10}{S_{B}}_{9}+o_{By2}S_{B9}S_{B10}-o_{Bz2}{C_{B}}_{9}}{-n_{Bx2}C_{B10}S_{B9}-n_{By2}S_{B9}S_{B10}+n_{Bz2}{C_{B}}_{9}})\\ \theta_{B7}= & \arctan2(\frac{n_{Bz2}{C_{B}}_{9}-n_{Bx2}C_{B10}S_{B9}-n_{By2}S_{B9}S_{B10}}{C_{B6}},a_{Bz2}C_{B9}-a_{Bx2}C_{B10}S_{B9}-a_{By2}{S_{B}}_{9}{S_{B}}_{10})\\ \theta_{B5}= & \arctan2(\frac{p_{By1}-d_{B1}a_{By1}}{d_{B3}},\frac{p_{Bx1}-d_{B1}a_{Bx1}}{d_{B3}})\\ \theta_{B4}= & \arctan2(\frac{p_{Bx1}C_{B5}+p_{By1}S_{B5}-d_{B1}(a_{Bx1}C_{B5}+a_{By1}S_{B5})}{d_{B3}},\frac{p_{Bz1}-d_{B5}-d_{B1}a_{Bz1}}{d_{B3}})\\ \theta_{B3}= & \arctan(\frac{a_{By1}C_{B5}-a_{Bx1}S_{B5}}{a_{Bx1}C_{B4}C_{B5}+a_{By1}C_{B4}S_{B5}-p_{Bz1}S_{B4}})\\ \theta_{B1}= & \arctan(\frac{o_{Bx1}C_{B5}{S_{B}}_{4}+o_{By1}S_{B4}S_{B5}+o_{Bz1}{C_{B}}_{4}}{-n_{Bx1}C_{B5}S_{B4}-n_{Bz1}{C_{B}}_{4}-n_{By1}S_{B4}S_{B5}})\\ \theta_{B2}= & \arctan2(\frac{a_{Bx1}C_{B4}C_{B5}-a_{Bz1}{S_{B}}_{4}+a_{By1}C_{B4}S_{B5}}{C_{B3}},a_{Bz1}C_{B4}+a_{Bx1}C_{B5}S_{B4}-a_{By1}{S_{B}}_{4}{S_{B}}_{5}) \end{array} \end{array}$$

**Table A3:** D-H parameters table of the links 1–5.

Rod *i*	*α_i_*_−1_ (°)	*a_i_*_−1_ (mm)	*d_i_* (mm)	*θ_i_* (°)
1	0	0	*d*_1_ = −58	*θ*_1_
2	−90	0	0	*θ*_2_
3	90	0	*d*_3_ = −125	*θ*_3_
4	−90	0	0	*θ*_4_
5	90	0	*d*_5_ = −90	*θ*_5_
